# Crystallization and visible–near-infrared luminescence of Bi-doped gehlenite glass

**DOI:** 10.1098/rsos.181667

**Published:** 2018-12-19

**Authors:** M. Majerová, R. Klement, A. Prnová, J. Kraxner, E. Bruneel, D. Galusek

**Affiliations:** 1 Department of Magnetometry, Institute of Measurement Science, Slovak Academy of Sciences, Dúbravská cesta 9, SK-842 19 Bratislava, Slovak Republic; 2 Centre for Functional and Surface Functionalized glass, Alexander Dubček University of Trenčín, Študentská 2, SK-911 50 Trenčín, Slovak Republic; 3 Vitrum Laugaricio – Joint Glass Center of the IIC SAS, TnU AD and FCHPT STU, Študentská 2, SK-911 50 Trenčín, Slovak Republic; 4 Department of Chemistry, Ghent University, Krijgslaan 281 S3, Gent 9000, Belgium

**Keywords:** gehlenite, flame synthesis, glass microspheres, photoluminescence properties, Bi^3+^-doped glasses

## Abstract

Gehlenite glass microspheres, doped with a different concentration of Bi^3+^ ions (0.5, 1, 3 mol%), were prepared by a combination of solid-state reaction followed by flame synthesis. The prepared glass microspheres were characterized from the point of view of surface morphology, phase composition, thermal and photoluminescence (PL) properties by optical and scanning electron microscopy (SEM), X-ray diffraction (XRD), differential scanning calorimetry (DSC) and PL spectroscopy. The closer inspection of glass microsphere surface by SEM confirmed a smooth surface. This was further verified by XRD. The basic thermal characteristics of prepared glasses, i.e. *T*_g_ (glass transition temperature), *T*_x_ (onset of crystallization peak temperature), *T*_f_ (temperature of the inflection point of the crystallization peak) and *T*_p_ (maximum of crystallization peak temperature), were estimated from the DSC records. High-temperature XRD experiments in the temperature interval range 600–1100°C were also performed. The PL emission properties of prepared glasses and their polycrystalline analogues (glass crystallized at 1000°C for 10 h) were studied in the visible and near-infrared (NIR) spectral range. When excited at 300 nm, the glasses, as well as their polycrystalline analogues, exhibit broad emission in the visible spectral range from 350 to 650 nm centred at about 410–450 nm, corresponding to Bi^3+^ luminescence centres. The emission intensity of polycrystalline samples was found to be at least 30 times higher than the emission of their glass analogues. In addition, a weak emission band was observed around 775 nm under 300 nm excitation. This band was attributed to the presence of a minor amount of Bi^2+^ species in prepared samples. In the NIR spectral range, the broad band emission was observed in the spectral range of 1200–1600 nm with the maxima at 1350 nm. The chemistry of Bi and its oxidation state equilibrium in glasses and polycrystalline matrices is discussed in detail.

## 1. Introduction

Since the 1990s, compounds with melilite structure have been intensively studied due to their interesting electrochemical [1], magnetic [2–4], luminescence [5–7] and structural properties [8–10]. The melilites are a large family of materials with the general formula ^[8]^A_2_^[4]^B^[4]^C_2_O_7_ where A is a large cation such as Ca, Sr, Ba, Na, K, Y, lanthanides, Pb or Bi; B is a small cation such as Al, Be, Co, Cu, Cd, Fe, Ga or Mg; and finally C = Si, B, Al, Cr, Ge or V. The number in square brackets [*N*] indicates the coordination number (CN) of the element. The structure of melilite compounds, which belong to a sorosilicate group of minerals, was first determined by Warren [11]. The structure has a tetragonal symmetry space group P-42_1_m and consists of BC_2_O_7_ layers parallel to (0 0 1) with corner-sharing BO_4_ and CO_4_ tetrahedra. The A cations are placed between these layers in distorted square antiprisms of oxygen atoms. Owing to their tetragonal and non-centrosymmetric crystal structure, lanthanides or transition metals can be accepted as dopants by the melilites [3].

Rare earth ion-doped melilite-type materials represented by gehlenite (Ca_2_Al_2_SiO_7_) have been intensively investigated over the past few decades. For example, gehlenite doped with Nd^3+^ ions is a good candidate for diode pumped laser, with a broad absorption around 806 nm [12]. Ca_2_Al_2_SiO_7_:Eu^3+^, Tb^3+^ as a potential candidate for phosphor converted light-emitting diodes was reported by Yang *et al*. [13]. Bernardo *et al*. compared amorphous Eu^3+^-doped gehlenite-based glass phosphor materials with their polycrystalline analogues [6]. It was determined that amorphous phosphor materials allow for more homogeneous dopant (or activator) distribution, due to the lack of grain boundaries to segregate and accumulate the dopants.

Recently, the optical properties of Bi-doped oxide glasses have been intensively investigated [14,15]. These glasses have interesting properties, such as simple colouring [16], third-order optical nonlinearity [17] and luminescence in the ultraviolet–visible wavelength range [18]. Compared to rare-earth-doped glasses, Bi-doped glasses exhibit ultra-broad infrared emission in the wavelength region from 1000 to 1700 nm with a full width at half maximum of the emission band up to about 300 nm. The bismuth-doped glasses, crystals and fibres thus have the potential to become the next generation of ultra-broadband optical amplifiers [19–28]. In materials (including glasses), Bi ions can be present in four oxidation states: Bi^+^, Bi^2+^, Bi^3+^ and Bi^5+^. As the melting temperature of the Bi-doped glasses increases, the bismuth valence state changes as follows:

$$Bi^{3+}\to Bi^{2+}\to Bi^{+}\to \mathrm{Bi},\;Bi_{2},\;Bi_{2}^{-},\;Bi_{3},\ldots \to {\left(Bi\right)}_{n}$$

where Bi_2_, Bi_2_^−^, Bi_3_, etc. are Bi clusters and (Bi)*_n_* represents metallic colloidal particles. The melting temperature, the glass composition, the atmosphere and the concentration of other polyvalent elements all strongly affect the reaction sequence [29].

The nature of active centres emitting in the near infrared (NIR) range is still controversial. Several hypotheses have been proposed, which can be summarized into three groups. The NIR luminescence is attributed alternatively to the presence of (i) bismuth with higher valence (Bi^5+^ and Bi^5+^O^2+^_n_ molecules) [23,30]; (ii) bismuth with lower valence (Bi^+^, neutral and negatively charged dimers—Bi_2_, Bi_2_^−^, Bi_2_^2−^) [20,31], the di- or tri-valent Bi ions are excluded, because it is generally known that Bi^2+^ and Bi^3+^ ions emit visible luminescence [15,18,32–34]; and (iii) point defects. To add to the confusion, changes of Bi activated NIR luminescence have been reported with the change of their local coordination. This indicates that NIR emission may be the result of the local structure of the active centres [14,23,35–37].

In this work, we investigated the luminescence properties of Bi_2_O_3_-doped gehlenite glass microspheres prepared by flame synthesis and studied the influence of crystallization of the glass on the luminescence in visible and NIR wavelength range.

## 2. Material and methods

Powder precursors for flame synthesis were prepared by solid-state reaction, from high-purity SiO_2_ (p.a., Polske odczynniki chemiczne, Gliwice), Al_2_O_3_ (p.a., Centralchem, Bratislava), Bi_2_O_3_ (99.9%, STREM Chemicals, USA) and CaCO_3_ (p.a., Centralchem, Bratislava). The compositions of prepared systems are summarized in table 1. At first, suitable amounts of the starting powders were weighed and homogenized in an agate mill in isopropyl alcohol for 4 h. After drying under an infrared lamp, the powders were calcined in a two-step process at 1000°C for 4 h in air. In the next step, the calcined powders were annealed at 1300°C for 4 h in a Pt crucible.

**Table 1.** Composition and temperature parameters of investigated glasses from DSC at 10°C min^−1^; *T*_g_—glass transition temperature, *T*_x_—crystallization onset temperature, *T*_f_—temperatures of the inflection points of the crystallization peak, *T*_p_—peak temperature of crystallization.

	composition (mol.%)										
sample	CaO	Al_2_O_3_	SiO_2_	Bi_2_O_3_	XRD quality	mean diameter (μm)	*T*_g_ (°C)	*T*_x_ (°C)	*T*_f1_ (°C)	*T*_f2_ (°C)	*T*_p_ (°C)
GBi0.0	50.00	25.00	25.00	0.0	amorphous	n.d.	860	970	980	996	994
GBi0.5	49.76	24.87	24.87	0.5	amorphous	7.1	863	978	989	1003	996
GBi1.0	49.50	24.75	24.75	1.0	amorphous	7.3	860	944	962	992	976
GBi3.0	48.50	24.25	24.25	3.0	amorphous	7.4	837	867 938	874 961	892 990	884 973

Glass microspheres were prepared from powder precursors by flame synthesis. The powders were fed into CH_4_–O_2_ flame with an estimated temperature of about 2800°C. The molten particles were quenched by spraying them with distilled water (to achieve a sufficient cooling rate to avoid crystallization), separated and dried. To eliminate any residual carbon from flame synthesis, the glass microspheres were calcined for 4 h at 650°C in air.

Primary information on the morphology of prepared microspheres was obtained by optical microscopy (Nikon ECLIPSE ME 600) in transmitted light at 10–50× magnification. More detailed examination of prepared glass microspheres was carried out by scanning electron microscopy (FEG SEM JEOL 7600F) at an accelerating voltage of 20 kV. The microspheres were fixed on an aluminium sample holder using conductive adhesive graphite tape and sputtered with gold (Carl Zeiss SC 7620 sputter coater) to prevent charging. For the SEM examination of a polished cross section of glass microspheres, the microspheres were embedded into a polymeric resin (Simplimet 1000, Buehler), carefully polished to prepare cross sections (Ecomet 300, Buehler) and sputtered with carbon to prevent charging.

The differential scanning calorimetry (DSC) measurements were carried out in the temperature range (30–1200°C) with the use of Netzsch STA 449 F1 Jupiter analyser. Nitrogen atmosphere (5.0 purity), heating rate (10°C min^−1^) and platinum crucibles with the sample mass ≈ 15 mg were used in the DSC experiments.

Phase compositions of prepared precursor powders and glass microspheres were studied by powder X-ray diffraction (XRD; Panalytical Empyrean, CuK*α* radiation, at ambient temperature in the *2θ* range of 10–80°). The crystallization properties of prepared microspheres were investigated by high-temperature XRD (HT XRD) using the same diffractometer equipped with a high-temperature cell (Anton Paar HTK 16), in the temperature range 600–1100°C in ambient atmosphere, and a heating rate of 10°C min^−1^.The software High Score Plus (v. 3.0.4, Pananalytical, The Netherlands) was used to evaluate diffraction data with the use of the COD 2017 database.

The photoluminescence spectra were recorded using Fluorolog FL3-21 spectrometer (Horiba Jobin Yvon) with Xe (450 W) arc lamp as an excitation source. The luminescence properties of Bi-doped gehlenite glasses were compared with their crystalline analogues obtained by isothermal crystallization of glass at 1000°C for 10 h under ambient atmosphere.

## 3. Results and discussion

**Figure 1.**
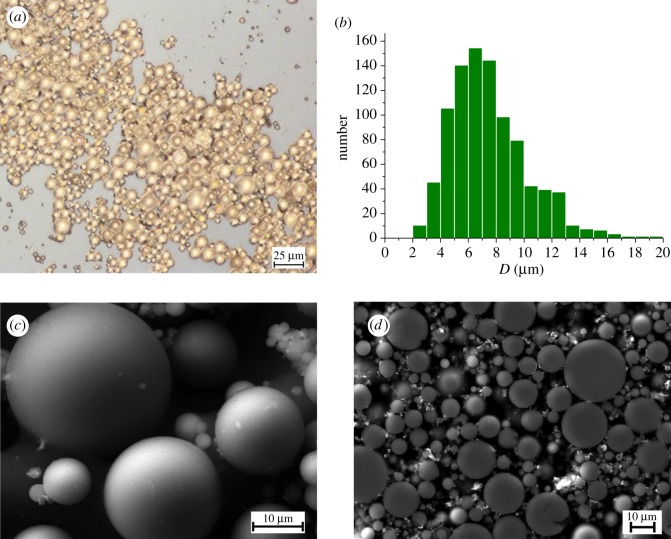
The results of optical microscopy and SEM examination and particle size distribution of prepared microspheres. (*a*) Optical micrograph of GBi0.5 microspheres, (*b*) particle size distribution of GBi3.0, (*c*) SEM image of as-prepared GBi3.0 microspheres, (*d*) SEM micrograph of a polished cross section of the GBi3.0 sample.

Preliminary inspection of prepared glasses using optical microscopy revealed that the glass particles were of spherical shape and were transparent in the visible wavelength region (figure 1*a*). Their diameter varied between 2 and 20 µm with a mean value of ≈7 µm similar for all prepared compositions. The representative particle size distribution is shown in figure 1*b*, with the predominant fraction of beads having a diameter in the range of 4 to 10 µm. More detailed examination of prepared glass microbeads was carried out by SEM. Representative SEM micrographs of microspheres are shown in figure 1*c,d*. Amorphous nature of glass microspheres was indicated by their smooth surfaces. SEM examination of polished cross sections confirmed the amorphous character of prepared glass microspheres; no morphological features indicating the presence of crystalline phases (e.g. facets, second phase inclusions) were observed (figure 1*d*).

**Figure 2.**
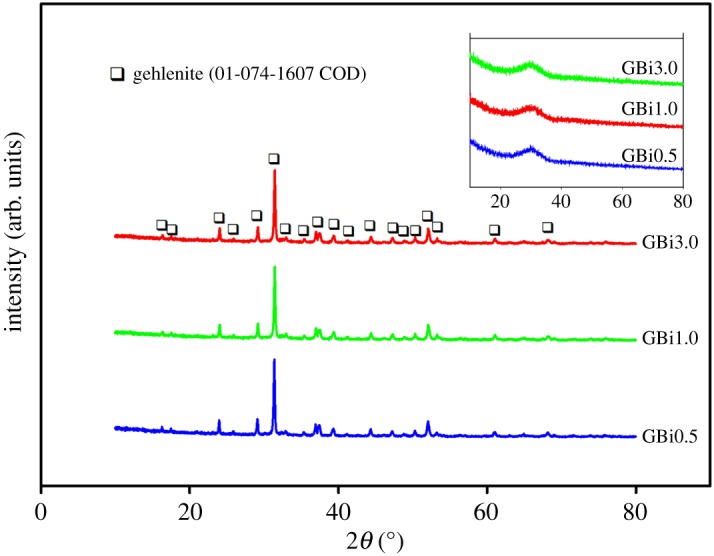
XRD patterns of crystallized microspheres (1000°C/10 h). The inset shows XRD patterns of Bi-doped gehlenite glassy particles after flame synthesis.

The phase composition of prepared glass microspheres was identified using XRD, as shown in the inset of figure 2. The absence of any discrete diffraction maxima and the presence of a broad band in the *2θ* range 24°–36° in XRD patterns of all prepared compositions confirmed their amorphous nature. The XRD patterns of microspheres crystallized at 1000°C for 10 h (figure 2) confirmed their polycrystalline character with the presence of pure gehlenite (01-074-1607 COD) as the only crystalline phase.

**Figure 3.**
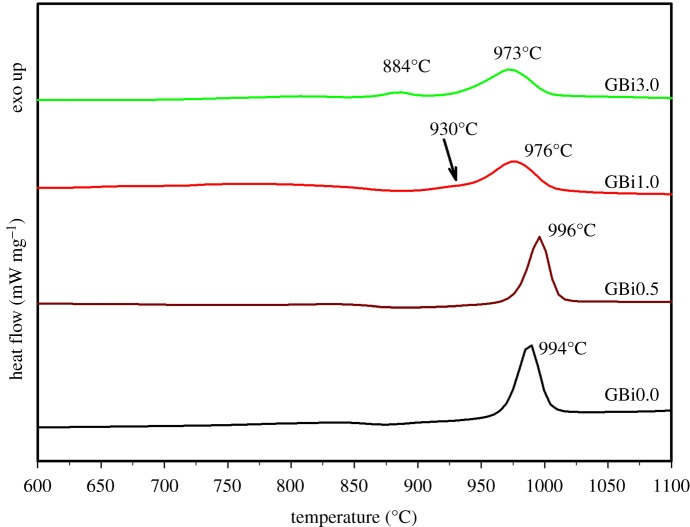
DSC records of prepared Bi-doped gehlenite glass microspheres.

Thermal properties of the prepared glasses were studied using DSC. DSC study of glass microspheres revealed significant differences in thermal behaviour of prepared systems. The DSC record of GBi0.5 sample contained one relatively broad exothermic peak centred at 996°C, which is close to the peak temperature of pure gehlenite glass prepared as microspheres, *T*_p_ approximately 994°C (figure 3). This exothermic effect was attributed to the crystallization of the gehlenite phase. Similar values around approximately 1000°C were also reported for gehlenite glass prepared by melt-quenching technique [38,39]. As the concentration of Bi_2_O_3_ in glass increases, the peak temperature is significantly shifted (about 20°C) to lower temperatures and broadened. For the sample GBi1.0, the DSC record contained one broad exothermic peak centred at 976°C; however, also the indication of another exothermic effect with the maxima at around 930°C was observed in the DSC trace. On the contrary, the DSC curve of GBi3.0 sample contained two broad exothermic peaks centred at 884°C and 973°C (figure 3), respectively.

The glass transition temperature (*T*_g_) and the onset of crystallization temperature (*T*_x_) were also estimated from the DSC records. In addition, the inflection points of crystallization peaks (*T*_f1_, *T*_f2_) were determined (the first derivatives of the DSC curves). The basic thermal characteristics of prepared glasses are summarized in table 1. The temperatures *T*_g_, *T*_x_ and *T*_p_ are shifted towards lower values with the increase in bismuth content in glass (table 1). As reported by Reddy *et al*. [40] in a study of melilite-type glasses with the addition of Bi_2_O_3_, the increase in Bi_2_O_3_ content may give rise to a decrease in the Si–O–Si bridging oxygen of the silicate structural units with progressive formation of non-bridging oxygens. This gradual depolymerization of the glass network well explains the observed decrease in *T*_g_ values from 863°C to 837°C. The lower values of *T*_x_ and *T*_p_ indicate the higher tendency of GBi1.0 and GBi3.0 glasses to crystallization in comparison with the GBi0.5. As Bi_2_O_3_ plays the role of network modifier [41], the increased tendency towards crystallization is most likely due to a decrease in viscosity of the glasses (partially depolymerized glass network), which may facilitate diffusion of cations. The decrease in *T*_g_ and *T*_p_ values with increasing Bi_2_O_3_ content was also reported by Kim *et al.* [41] for the glasses in the system Bi_2_O_3_–B_2_O_3_–SiO_2_. Moreover, Bi_2_O_3_ may act as an effective nucleating agent, thus leading to the decrease in nucleation/crystallization temperature and unusual thermal behaviour of GBi1.0 and GBi3.0 glasses. The crystallization process, however, seems to be more complex and further detailed kinetic study is required.

**Figure 4.**
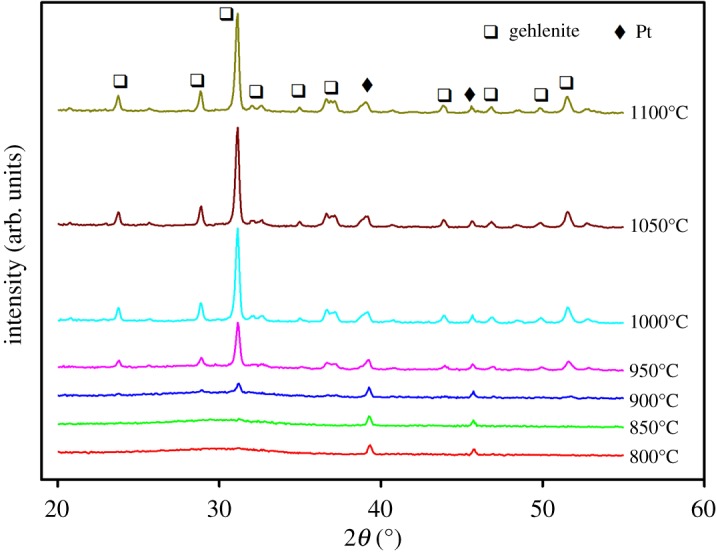
HT XRD records of the GBi3.0 microspheres recorded at various temperatures.

**Figure 5.**
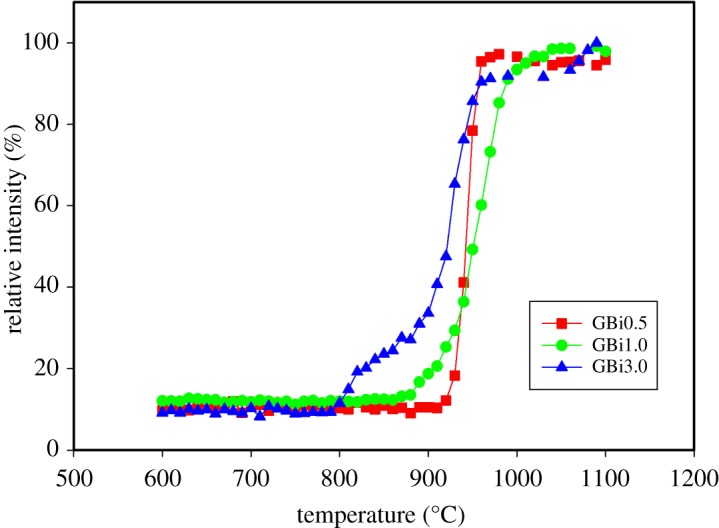
Temperature dependences of relative intensities of the <1, 2, 1> gehlenite diffraction peak.

**Figure 6.**
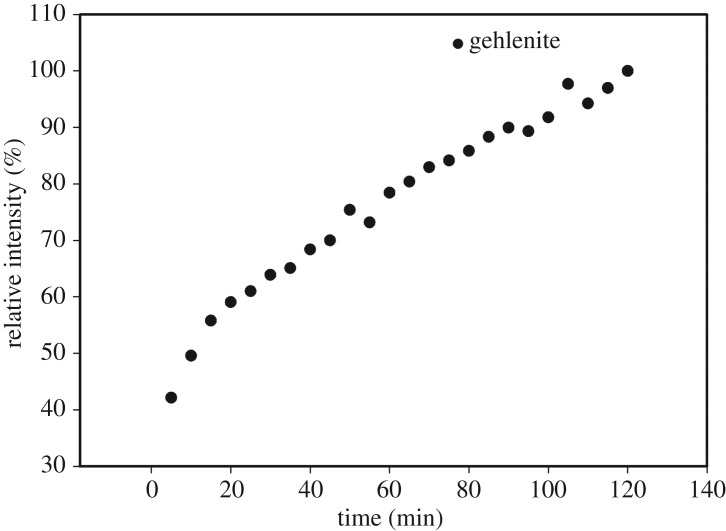
The time dependence of the relative intensity of the <1, 2, 1> gehlenite peak during isothermal heating at 884°C.

To determine the origin of the first peak in the DSC record of GBi3.0 glass and to better understand thermal behaviour of prepared samples, HT XRD experiments were performed and phase evolution with temperature was studied. HT XRD experiments were carried out in the temperature interval 600–1100°C, at the heating rate of 5°C min^−1^, and with an XRD pattern recorded every 10°C. Figure 4 shows selected diffraction patterns measured during the non-isothermal HT XRD experiments. Crystallization of only gehlenite phase (01-074-1607 COD) was observed in the whole measured temperature interval, with the onset of crystallization at 800°C for GBi3.0 sample, 870°C for GBi1.0 and 920°C for GBi0.5. These temperatures correspond to the temperature at which the most intense peak of the gehlenite phase (<1, 2, 1>) was first observed in the XRD trace and raised. The results obtained are in good agreement with the results of DSC analysis. From the diffraction patterns recorded at different temperatures, the temperature dependences of the relative intensity of the most intense gehlenite peak at 2*θ* = 31.394° were determined (figure 5) and temperature intervals with the most pronounced increase in relative intensity of the <1, 2, 1> gehlenite peak were estimated. These were found to be as follows: (800–820)°C and (930–940)°C for the GBi3.0 glass, (960–970)°C for GBi1.0, and (940–950)°C for GBi0.5. The presence of two temperature intervals with the highest increase in relative intensity of the <1, 2, 1> gehlenite peak in GBi3.0 corresponds to the presence of two peaks in DSC record and suggests crystallization of gehlenite phase in two steps. Figure 6 also indicates that the crystallization of GBi0.5 glass is completed at temperature ≈ 990°C, and for GBi1.0 and GBi3.0 glasses at approximately 1040°C. Above this temperature only negligible growth of relative intensity of the <1, 2, 1> gehlenite peak was observed.

Based on the non-isothermal HT XRD and DSC analysis, an isothermal HT XRD experiment was performed with the GBi3.0 glass. The glass microspheres were heated to 884°C and one XRD pattern was recorded every 5 min with a total duration of isothermal heating of 240 min. The results of this experiment showed crystallization of only one phase—gehlenite (01-074-1607 COD). Time dependence of the relative content of gehlenite phase in isothermally annealed GBi3.0 sample is shown in figure 6. After a fast initial increase in the content of crystalline gehlenite in the first 20 min, the crystallization slowed down, and the content of crystalline gehlenite increases approximately linearly until the end of the experiment.

**Figure 7.**
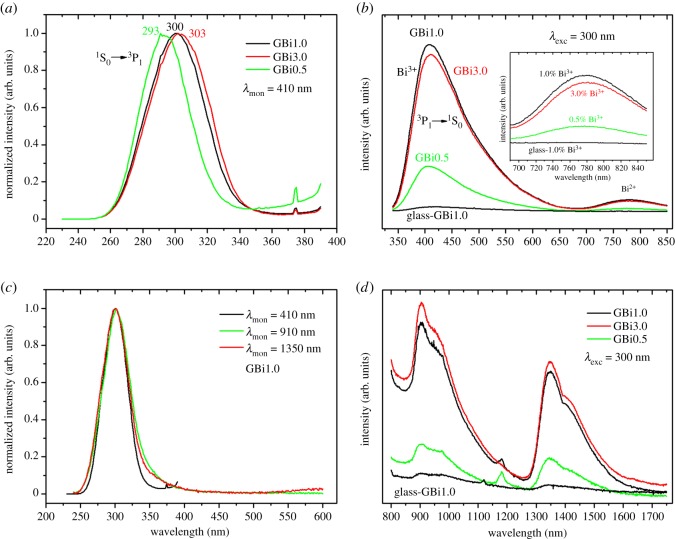
The photoluminescence excitation (*a,c*) and emission spectra (*b,d*) of Bi^3+^-doped crystallized microspheres.

The photoluminescence (PL) emission properties of prepared glasses and crystalline analogues (glass crystallized at 1000°C for 10 h) were studied in the visible and NIR spectral range. The excitation and emission spectra of prepared samples are shown in figure 7. Owing to the very low PL emission intensity of glasses compared to the polycrystalline samples, the discussion is mainly focused on the PL properties of polycrystalline samples.

The Bi^3+^ ion has 6s^2^ electron configuration and thus the ground state is ^1^S_0_, and 6s6p configuration in the excited state, which gives rise to the triplet levels ^3^P_0_, ^3^P_1_, ^3^P_2_ and singlet state ^1^P_1_, in order of increasing energy. According to the dipole selection rules, excitations usually occur from the ^1^S_0_ ground state to the ^3^P_1_ and ^1^P_1_ states [42]. The excitation spectra (*λ*_mon_ = 410 nm) exhibit broad absorption band centred at about 300 nm, corresponding to ^1^S_0_ → ^1^P_1_ transition of Bi^3+^, that is slightly red shifted as the concentration of Bi^3+^ ions increases (form 293 to 303 nm); figure 7*a*. When excited at 300 nm, the glasses as well as their crystalline analogues exhibit broad emission band in the visible spectral range from 350 to 650 nm centred at about 410–450 nm, corresponding to ^3^P_1_ → ^1^S_0_ transition within the Bi^3+^ luminescence centres. Similar broadband emission was observed by Li *et al*. [5] in charge non-compensated and alkali ion charge compensated Bi^3+^ doped gehlenite, obtained by solid-state reaction. The blue emission intensity of crystalline samples is high and was found to be at least 30 times higher than the emission of the glass samples with colour coordinates at (*x*, *y* = 0.196, 0.191). The PL intensity increases with increasing Bi^3+^ concentration. However, for sample with 3% of Bi^3+^, the intensity is lowered thus indicating that concentration quenching may operate at this doping level.

In addition, a weak emission band was observed around 775 nm under 300 nm light excitation. This band is due to the presence of Bi^2+^ species in prepared samples. The electron configuration of Bi^2+^ is 6s^2^6p^1^ with ^2^P_1/2_ ground state and ^2^P_3/2_ as the first excited state. This excited state can be further separated by crystal field splitting into two sublevels ^2^P_3/2_(1) and ^2^P_3/2_(2), in order of increasing energy. In fact, the transition ^2^P_3/2_(1) → ^2^P_1/2_ is responsible for emission that is usually observed in the orange–red spectral range (emission maxima 600–700 nm) [43]. The shift of this emission found in our Bi-doped samples to the deep red spectral range indicates the strong crystal field splitting of the ^2^P_3/2_ states.

In the NIR spectral region, two broad band emissions were observed in the spectral ranges of 850–1200 and 1200–1600 nm with the maxima at 905 and 1350 nm, respectively (figure 7*c,d*). The first emission is superposed on the deep red emission originating from ^2^P_3/2_(1) → ^2^P_1/2_ transition of Bi^2+^ ions. The concentration dependence of NIR emission intensity follows the same order as described above for visible range emission. The origin of these NIR emissions is still a matter of dispute; however, many authors ascribe this transition to the lower oxidation state of the bismuth, such as Bi^+^, Bi^0^ or cluster ions [44]. Thus, it is reasonable to expect that the observed NIR emissions originate from Bi^+^ ions incorporated in the gehlenite crystal host.

**Figure 8.**
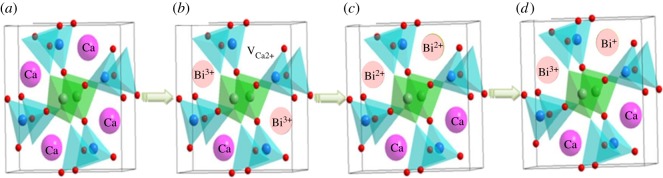
(*a*) The Ca_2_Al_2_SiO_7_ host structure; (*b*) the incorporation of the Bi^3+^ ions; (*c,d*) the incorporation of the Bi^2+^ ions; the incorporation of the Bi^3+^ and Bi^+^ ions into the host structure for charge compensation [5].

The structure of gehlenite (Ca_2_Al_2_SiO_7_) is schematically depicted in figure 8*a*. The Ca atoms are closely surrounded by (Al/Si)O_7_ and AlO_4_ polyhedra creating a porous structure. Ca^2+^ in the tetragonal Ca_2_Al_2_SiO_7_ is coordinated with eight O^2−^ atoms, forming a distorted polyhedron. When Bi^3+^ is doped into Ca_2_Al_2_SiO_7_, it tends to substitute for calcium rather than aluminium or silicon sites as a result of matched ionic size (note that for Bi^3+^ with CN of 8, R_Bi3+,CN=8_ = 1.17 Å; and R_Ca2+,CN=8_ = 1.12 Å; R_Al3+,CN=4_ = 0.39 Å; R_Si4+,CN=4_ = 0.26 Å). There is, however, a charge imbalance between Bi^3+^ and Ca^2+^ ions. In general, the charge imbalance is induced by creation of internal defects, for instance, negatively charged Ca^2+^ vacancies or positively charged O^2−^ vacancies. These internal structural defects often lead to quenching of luminescence due to the energy transfer from luminescence centres to defects. Figure 8*b* demonstrates such a situation, when 3Ca^2+^ ions have been replaced by 2Bi^3+^ ions, resulting in a Ca^2+^ vacancy: 3Ca^2+^ = 2Bi^3+^ + V_Ca2+_. In order to compensate the charge defect, the M^+^ ion (e.g. alkali ions Li^+^, Na^+^, K^+^) should be introduced as the charge compensation or the Bi^3+^ ion should change its oxidation state to Bi^2+^ and/or Bi^+^ (figure 8*c,d*). This is most likely the case in samples studied in this work, as documented by PL emissions originating from three different bismuth sites, Bi^3+^, Bi^2+^ and Bi^+^, respectively. All three bismuth oxidation states were also observed in PL spectra of the prepared glass samples, however, with much lower emission intensity.

## 4. Conclusion

Three Bi-doped gehlenite precursor powders with different content of Bi_2_O_3_ (0.5, 1, 3 mol%) were prepared by a standard solid-state reaction method. X-ray amorphous glass microspheres, with diameters up to 25 µm and transparent in visible light, were then prepared from the precursor powders by flame synthesis. The addition of various amounts of Bi has a significant effect on the thermal properties of prepared glass microspheres:

1. For the compositions with the lowest Bi content, only one exothermic effect was observed corresponding to the crystallization of gehlenite phase. In the case of a sample containing 1.0 mol% of Bi_2_O_3_, the DSC record contains one broad exothermic peak but with an indication of another exothermic effect. Two exothermic maxima were found on the DSC curve of the GeBi3.0 glass.
2. The glass transition temperature and the onset of crystallization temperature of gehlenite glasses decrease with increasing content of Bi in the samples.
3. The HT XRD results confirmed the crystallization of gehlenite over the entire temperature range indicating that it was a two-step gehlenite crystallization in the case of GBi3.0 glass. The crystallization mechanism of gehlenite phase in GBi1.0 and GBi3.0 glasses is not a trivial one and detailed study of crystallization kinetics is required to clarify this effect.

The PL emission properties of prepared glasses and their crystalline analogues (glass crystallized at 1000°C for 10 h) were studied in the visible and NIR spectral range. The emission intensity of crystalline samples was found to be at least 30 times higher than the emission of the glass analogues. The three types of PL emissions in different spectral regions (visible, deep red and NIR range) revealed the simultaneous presence of bismuth ions in three oxidation states Bi^3+^, Bi^2+^ and Bi^+^, with the last two oxidation states stabilizing the host structure and compensating charge imbalance between the Bi^3+^ and Ca^2+^ ions. This structural arrangement favours replacement of Ca^2+^ in the crystal host and, hence, strong luminescence emission from Bi^3+^.

## Data accessibility

All data are included in the paper.
